# Multiplex Detection of Magnetic Beads Using Offset Field Dependent Frequency Mixing Magnetic Detection

**DOI:** 10.3390/s21175859

**Published:** 2021-08-31

**Authors:** Ali Mohammad Pourshahidi, Stefan Achtsnicht, Mrinal Murali Nambipareechee, Andreas Offenhäusser, Hans-Joachim Krause

**Affiliations:** 1 Institute of Biological Information Processing-Biolelectronics (IBI-3), Forschungszentrum Jülich, 52425 Jülich, Germany; a.pourshahidi@fz-juelich.de (A.M.P.); achtsnicht@fh-aachen.de (S.A.); mrinalmurali@gmail.com (M.M.N.); a.offenhaeusser@fz-juelich.de (A.O.); 2 Faculty of Mathematics, Computer Science and Natural Sciences, RWTH Aachen University, 52062 Aachen, Germany; 3 Institute of Nano- and Biotechnologies (INB), FH Aachen University of Applied Sciences, 52428 Jülich, Germany

**Keywords:** magnetic nanoparticles, frequency mixing magnetic detection, multiplex detection, colorization

## Abstract

Magnetic immunoassays employing Frequency Mixing Magnetic Detection (FMMD) have recently become increasingly popular for quantitative detection of various analytes. Simultaneous analysis of a sample for two or more targets is desirable in order to reduce the sample amount, save consumables, and save time. We show that different types of magnetic beads can be distinguished according to their frequency mixing response to a two-frequency magnetic excitation at different static magnetic offset fields. We recorded the offset field dependent FMMD response of two different particle types at frequencies *f*_1_ + *n*⋅*f*_2_, *n* = 1, 2, 3, 4 with *f*_1_ = 30.8 kHz and *f*_2_ = 63 Hz. Their signals were clearly distinguishable by the locations of the extremes and zeros of their responses. Binary mixtures of the two particle types were prepared with different mixing ratios. The mixture samples were analyzed by determining the best linear combination of the two pure constituents that best resembled the measured signals of the mixtures. Using a quadratic programming algorithm, the mixing ratios could be determined with an accuracy of greater than 14%. If each particle type is functionalized with a different antibody, multiplex detection of two different analytes becomes feasible.

## 1. Introduction

Usage of superparamagnetic magnetic beads (MBs) in various biomedical branches has increased over the years [1,2]. They are used, for example, as tracer agents in imaging techniques [3,4], carriers in drug delivery [5,6], and especially as markers in biosensors [7,8]. In the area of biosensing, magnetic beads are used as a separation tool [9] or as markers [10]. Detection of these particles can be achieved by different methods such as susceptometry [11], relaxometry [12], and frequency mixing magnetic detection [13].

Frequency mixing magnetic detection (FMMD) has proven to be very selective for superparamagnetic particles and thus it has been successfully used for detection of a variety of biological targets. For example, recently, the quantification of Aflatoxin B1 [14] and of different antibiotics in milk [15] has been shown using a competitive magnetic immunoassay. Furthermore, by employing a noncompetitive sandwich immunoassay method, the detection of cholera toxin subunit B [16], Francisella tularensis [17], C-reactive protein [18], plant viruses [19], and influenza viruses [18,19,20] has been shown.

In the last decade, there has been a widespread interest in multiplex detection that is the ability to detect more than one biological target within a single sample since it can be beneficial in terms of time and financial expenditure as well as reducing the needed sample amount. Different approaches have been taken, for example, lateral flow biosensors were used for multiple analyte detection through fluorescent and colorimetric methods for detection of viruses [21], bacteria [22], and antibodies [23]. In [24], a magnetic relaxation switch approach was used for detection of multiple analytes. Moreover, multiplex detection of protein assays was performed using GMR sensors [25].

Furthermore, susceptibility based measurement techniques have also been able to simultaneously detect different magnetic nanoparticles and beads. For example, simultaneous detection of small size particles was completed using the AC susceptometry technique [26,27,28]. On the other hand, the frequency mixing magnetic detection approach has also shown promising results in terms of multiplex detection; for instance, it was shown that multiplex detection can be performed by spatial separation using 3D printed modular immunofiltration columns [29] and also using the amplitude and the phase of the mixing harmonics one can differentiate among different types of magnetic beads [30,31,32,33]. These publications emphasized the fact that the phase response of the magnetic nanoparticles is type specific and can be utilized for multiplex detection.

Lenglet [34] showed that different types of magnetic particles can be distinguished according to the offset field dependence of their frequency mixing responses. This technique has been shown to be applicable to multiparametric detection [35]. The mixing ratio of two types of magnetic particles with different hydrodynamic diameters has been determined from the ratio of the fifth to third harmonic response amplitude [26]. The distinction is based on the particles’ different structural magnetic properties [36]. Another approach to particle distinction is based on their differences in Brownian relaxation time constants, which enables discrimination based on two-frequency measurements [37] or multichannel image reconstruction [38,39].

In this work, we report on further developments in multiplex detection using the FMMD technique by employing a static magnetic offset field and present a method for determining the percentages of the constituents of a binary particle mixture from their measured frequency mixing response.

## 2. Materials and Methods

### 2.1. Magnetic Beads

In this study, we utilized different types of commercial superparamagnetic beads procured from micromod Partikeltechnologie GmbH, Rostock, Germany. Bead sizes ranged from 50 nm to 1000 nm in hydrodynamic diameter (*d_h_*). The superparamagnetic beads were selected from both plain and surface functionalized beads. For the smaller sized beads, SynomagD particles incorporating nanoflower-shaped cores with *d_h_* of 50 nm having a plain surface (Product code: 104-00-501) with a concentration of 25 mg/mL, and with *d_h_* of 70 nm, surface-functionalized with streptavidin (Product code: 104-19-701) with a concentration of 5 mg/mL were used. Furthermore, streptavidinated Perimag beads with *d_h_* of 130 nm (Product code: 102-19-132) having a concentration of 5 mg/mL and Nanomag CLD beads with *d_h_* of 300 nm (Product code: 05-19-302) with a concentration of 10 mg/mL were used. Additionally, for experiments with larger beads, streptavidinated Nanomag-CLD/Synomag-CLD with *d_h_* of 1 µm (Product code: 05-19-505) having a concentration of 10 mg/mL and SynomagD with *d_h_* of 1 µm (Product code: 104-19-103) with a concentration of 10 mg/mL were used.

### 2.2. Sample Preparation

For analyte detection using magnetic beads as quantifiers through the FMMD, a noncompetitive or competitive immunoassay procedure can be used. In the latter approach, the analyte is captured by an immobilized antibody and then marked with magnetic beads. Since this study intends to investigate the properties and responses of the pure superparamagnetic beads, a complete immunoassay was not performed. Instead, the beads were bound to immunofiltration columns directly in order to inhibit Brownian relaxation and only retain Néel relaxation. Thus, the measurement conditions were similar to the case when a complete immunoassay is performed with the advantage of excluding biological variation.

Magnetic beads of different types were immobilized on equilibrated abicap HP columns procured from Senova Gesellschaft für Biowissenschaften und Technik mbH, Weimar, Germany. The equilibration process of the filters was performed according to [19]. The immobilization process was performed by pouring the superparamagnetic bead solutions over the filters and letting them run through by gravity flow. The unbound beads were then removed from the filter by washing the columns twice using 500 µL of distilled water. The concentrations were all based on the magnetic bead concentration in their stock solution provided by the manufacturer.

For the experiment involving samples made from the mixture of different beads, a binary mixture was prepared from two different bead types with volume ratios of 0%: 100%, 25%: 75%, 50%: 50%, 75%: 25% and 100%: 0%, maintaining the total volume of the bead solution at 10 µL. After mixing with a vortexer, the solution was further diluted in 400 µL of distilled water pipetted over the abicap column and then immobilized on the equilibrated filters. The detailed explanation is given in Section 3.3.

### 2.3. Frequency Mixing Magnetic Detection

Frequency mixing magnetic detection (FMMD) is a method based upon the intermodulation of two distinct alternating magnetic fields with low and high-frequency spectral components [13]. The resulting mixing harmonics are measured and used to selectively quantify the ensemble of superparamagnetic nanoparticles.

The magnetization of these particles is described by the expression [13]:

(1) $$M\left(B\right)=M_{s}\cdot ℒ\left(\frac{m_{p}B}{k_{B}T}\right)$$

where *M_s_* is the saturation magnetization of the magnetic material and $m_{p}=M_{s}\frac{\pi }{6}d_{c}^{3}$ is the saturation magnetic moment of a single particle of core diameter *d_c_*. *B* denotes the applied magnetic field, *k_B_ T* is the product of Boltzmann constant and absolute temperature, and $ℒ\left(\xi \right)=coth\;\xi -\frac{1}{\xi }\;$ denotes the Langevin function used to describe the magnetization behavior of superparamagnetic materials [40].

**Figure 1:**
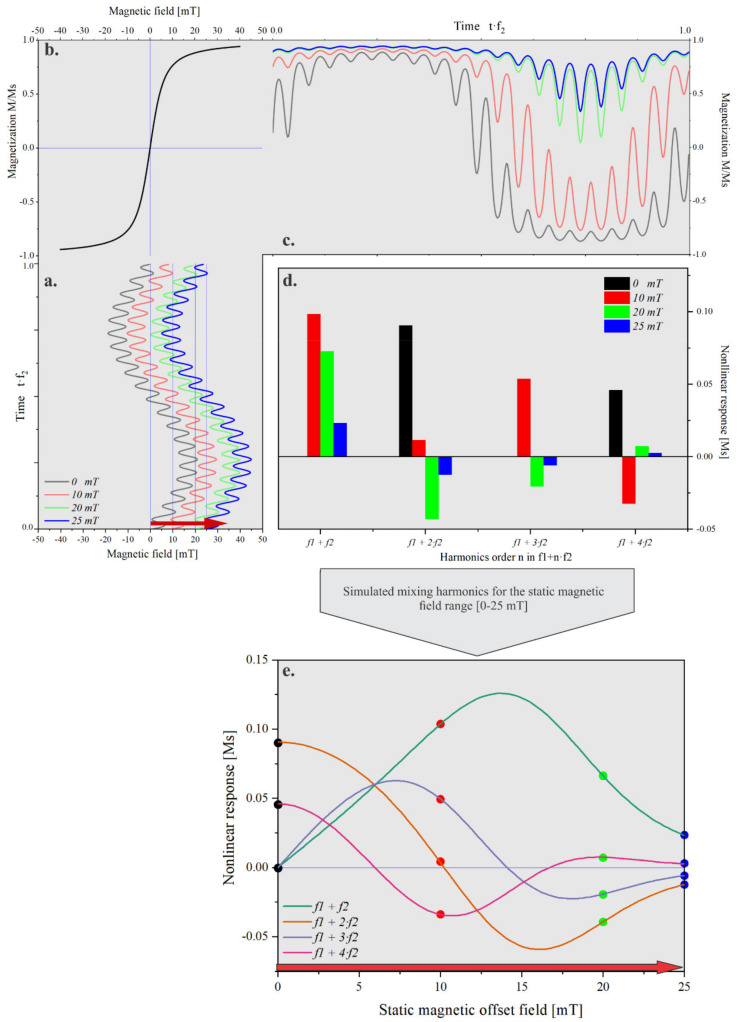
Two frequency magnetic excitation fields (*f*_1_, high frequency and *f*_2_*,* low frequency) (**a**) were applied to the ensemble of superparamagnetic magnetic nanoparticle with a magnetic moment of 200 kµB (**b**). The response of the particles was obtained from their nonlinear magnetization containing even and odd frequency mixing harmonics (**c**). In the absence of a static magnetic offset field, only odd harmonics appeared. Even harmonics emerged upon introducing a static magnetic offset field *B*_0_ (**d**). The nonlinear response traces showed specific features upon variation of the static magnetic offset field, such as maxima, minima, and zero crossings (**e**).

In this technique, as illustrated in Figure 1, the superparamagnetic beads were subjected to two alternating magnetic fields denoted as the driving field and excitation field. With sufficient strength within the range of a few mT, the driving field having a frequency (*f*_2_) in the range of a few tens of Hertz drives the magnetization of the superparamagnetic beads into the vicinity of a saturation level. On the other hand, the excitation field with the frequency (*f*_1_) is used to probe the magnetization state of the superparamagnetic beads.

In this research, the excitation field was set to have a frequency of *f*_1_ = 30.786 kHz with a magnetic flux density of *B*_1_ = 0.31 mT and the driving field was set to have a frequency of *f*_2_ = 62.95 Hz with a magnetic flux density amplitude of *B*_2_ = 16.4 mT. Thus, the time depended magnetic field has the form of

(2) $$B\left(t\right)=B_{0}+B_{1}sin\left(2\pi f_{1}t\right)+B_{2}sin\left(2\pi f_{2}t\right)\;$$

where $B_{0}$ is the static magnetic offset field which is varied from 0 to 24 mT in steps of 0.48 mT using an electromagnet connected to a constant current source.

From the resulting magnetization response, the even and odd mixing harmonics at frequencies *f*_1_ *± n·f*_2_ were demodulated and used for the detection of the particle signal and for the identification of the particle types. In the absence of an offset magnetic field, only odd mixing terms appear, thus *n* would be an even integer. In case of a nonvanishing static offset field, the odd mixing terms such as the second and fourth-order mixing frequencies (*n* = 1, 3) also appear. In the case of small excitation amplitudes, the frequency mixing harmonics do follow the derivatives of the Langevin function obtained through the Taylor approximation [13]. For larger excitation fields, the frequency mixing harmonics, as depicted in Figure 1e, can be numerically calculated using Equations (8) and (9) of Ref. [41].

Through variation of the static offset magnetic field, the nonlinear frequency mixing harmonic response of the magnetic nanoparticles exhibited characteristic points (maxima, minima, and zero-crossings), henceforth called features.

### 2.4. Static Magnetic Offset Setup

The appearance of the odd mixing frequencies as mentioned earlier in Section 2.3 can only occur in conjunction with a nonvanishing static magnetic offset field.

**Figure 2:**
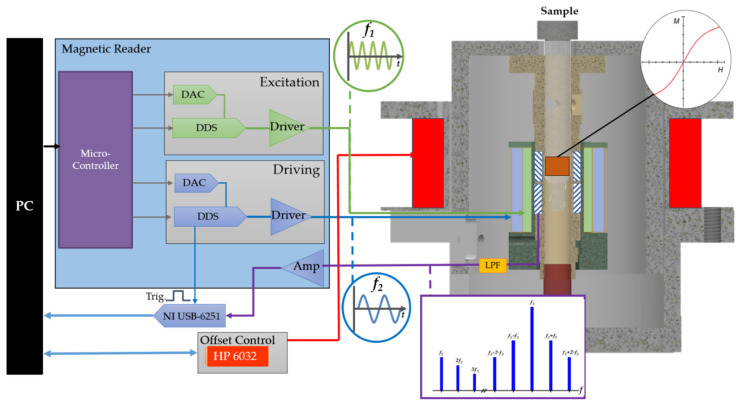
Schematic overview of the magnetic frequency mixing detection setup with the static magnetic offset field. A PC was used for controlling the magnetic reader and measurement. The magnetic reader consisted of a microcontroller, two direct digital synthesis (DDS) chips, and digital to analogue convertor (DAC) filters and drivers supplying the excitation and driver coils. A cutoff schematic of the measurement head is shown with excitation (green), driving coil (blue), and the static offset coil (red). The static offset coil was controlled by an HP 6032 power supply. The output of the detection coil, which contained the information about the mixing frequencies was connected via a low pass filter (LPF) to a preamplifier (Amp), which was built into the reader, and then to a National Instrument measurement card USB-6251 for a triggered measurement.

The measurement setup used in this study for detection and evaluation of the nonlinear mixing harmonics comprised a magnetic reader described in [16,29,30], a measurement head housing the excitations and detection coils, and an offset electromagnet placed around the measurement head driven by a programmable current source HP 6032 from Hewlett Packard to generate the static offset magnetic field. Additionally, a National Instrument measurement card (NI USB-6251) and a PC were utilized for data acquisition. The magnetic reader and the measurement card were both controlled through custom-built software developed in LABVIEW 2016. The simplified block diagram of the experimental setup together with a crosssectional sketch of the measurement head is shown in Figure 2.

The offset generating electromagnet was wound around an aluminum bobbin with an inner diameter of 64 mm and a height of 25 mm. The coil was made from 320 windings in 14 layers from copper wire having a nominal diameter of 1 mm resulting in a resistance of 1.8 Ω. For determination of the coil factor (in µT/mA), different currents were applied using a constant current source DIGISTANT 6425 T from Burster Präzisionsmeßtechnik, Gernsbach, Germany, and the resultant magnetic field was measured by a Fluxgate Fluxmaster from S. Mayer Messgeräte Münster, Germany. The relation was then determined using a linear fit to the measured data, yielding a coil factor of 4.8 μT/mA.

To test the linearity of the system over a larger range and also to include the effects of the later used HP 6032 Power Supply, an additional measurement was carried out using an uncalibrated 912 Gaussmeter from RFL. For these measurements, the power supply was used in the current control mode. The current was set from 0 to 7.0 A in steps of 0.25 A. It was found that the linear fit had an adjusted R² of 0.99.

To make sure that the coil operated safely without overheating, a watercooling strategy was used. A silicone tube with an outer diameter of 5 mm and wall thickness of 1 mm was wound around the coil and connected to a water cooling set “Alphacool NexXxoS Cool Answer 240 LT/ST” from Alphacool International GmbH (Braunschweig, Germany). Temperatures were monitored using two digital temperature sensors of type DS18B20 from Maxim Integrated, San Jose, California, placed on the outside of the water cooling pipes and on top of the aluminum coil body. They were connected via an Arduino to the PC where the values were recorded.

It was important to record the measurement signal with and without the sample in order to efficiently suppress background signals which may arise due to spurious frequency mixing in the readout electronics and nonlinear effects arising from the pickup coils. To reduce user interaction, an automatized insertion and removal of the samples throughout the measurement was implemented. A servo motor was used to operate an in-house fabricated rod made out of Polyvinyl chloride (PVC) that automatically lifted the sample during background measurement. Through this mechanism, the sample was always brought into the optimal measurement position and removed for background signal measurement afterwards.

### 2.5. Measurement Procedure

#### 2.5.1. Measurement Protocol

The measurement device was warmed up until the temperature and the measurement signal were stabilized. The scanning procedure then commenced in an automatized fashion. Initially, the background signal was measured for three and half minutes while the sample was kept outside the measurement head. Then the sample was placed into the measurement position using the servo, as explained in the experimental setup section. The sample was measured for three and half minutes and then pushed out. The static magnetic offset field was then changed to the next value and the same procedure was repeated until the final step of the magnetic field strength was reached.

#### 2.5.2. Data Processing

Data analysis and calculations were completed using scripts developed using python programming language. During the data processing, initially, background correction was performed by subtracting the reference background signal from the measurements and in the later stage, the frequency-dependent phase shift was corrected by performing a linear fit to the measured data in the complex plane and calculating the projection of the measurement points to that fit line.

The interpolation program to determine the concentration of the mixed samples based on the quadratic programming approach was developed in LabVIEW 2016 environment and the graphical representation was performed using OriginPro 2019.

The unit conversion of the signal amplitude from mV to a nonlinear magnetic moment in nAm² was performed by obtaining a calibration factor according to the method presented in [41] for calibration of magnetic reader sensitivity in case of the digital demodulation.

### 2.6. Quadratic Programming Optimization

Determination of the contributing number of beads in a two-bead mixture measurement was achieved by finding the linear combination of single particle reference measurements that minimized the quadratic deviation to the mixture measurement. For a binary mixture, the individual measurements *Ref_A_* and *Ref_B_* of both particle types A and B are multiplied with coefficients *x_A_* and *x_B_* and added. The set of coefficients that minimizes the quadratic deviation between the mixture measurement and the approximation *x_A_⋅Ref_A_* + *x_B_⋅Ref_B_* is determined. Hence, the nonlinear magnetic moment response of the magnetic beads over a range of static offset field is used for this purpose.

These measured reference signal values are called *Ref_i,j_* where the index *i* stands for the particle type which was measured, and the index *j* denotes the static magnetic offset field *B_j_* at which measurement data *M_j_* are being acquired. It turned out that parameter estimation was significantly improved when it was extended over the first four harmonics *f*_1_ *+ n⋅f*_2_ (*n =* 1, 2, 3, 4) simultaneously, instead of using data from only one harmonic. For that purpose, index *j* enumerates all measurement points. In our case, it simply extended sequentially over all four harmonics, so it counted along the *B*_0_-axis of *f*_1_ *+ f*_2_, continued along the *B*_0_-axis of *f*_1_ *+* 2⋅*f*_2_, until it ended at the highest field value of *f*_1_ *+* 4⋅*f*_2_.

In the case of relatively small concentrations of magnetic nanoparticles in solution, the particle–particle interaction can be neglected [41,42]. Therefore, it can be assumed that all particles contribute individually to the total measured signal, so the total signal can be calculated as a linear combination of the signals of all constituents of the sample. Therefore, the total reference measured signal *Ref_j_* at a static field value *B_j_* can be written as a weighted sum

(3) $$Ref_{j}=\underset{i}{\sum }x_{i}\cdot Ref_{i,j}\;$$

over the reference signals of different particles type *i* with weights *x_i_*. These amounts *x_i_* of type *i* particles are the unknowns to be determined, representing the contribution of each particle type to the overall signal.

For performing the optimization procedure, the sum *S* of the square residuals between measurement and reference should be minimized, i.e.,

(4) $$S=\underset{j}{\sum }{\left[M_{j}-\left(\underset{i}{\sum }x_{i}\cdot Ref_{i,j}\right)\right]}^{2}\to min.$$

Multiplying out *S* yields the following quadratic form to be minimized:

(5) $$S=\underset{j}{\sum }\left(\underset{i}{\sum }x_{i}\cdot Ref_{i,j}\right)\cdot \left(\underset{k}{\sum }x_{k}\cdot Ref_{k,j}\right)-2\cdot \underset{j}{\sum }M_{j}\cdot \left(\underset{i}{\sum }x_{i}\cdot Ref_{i,j}\right)+\underset{j}{\sum }M_{j}^{2}\to min.$$

This can be rewritten in the standard form used in the so-called quadratic programming (QP) [43],

(6) $$S=2\left(\frac{1}{2}\;{\vec{x}}^{T}\cdot Q\cdot \vec{x}+{\vec{c}}^{T}\cdot \vec{x}\right)+\underset{j}{\sum }M_{j}^{2}\to min.$$

with a symmetric *n* × *n*, positive definite matrix *Q* (*n* is the number of reference measurement curves, enumerated by index *i* = 1, …, *n*)

(7) $$Q=\left[\begin{array}{cccc} \sum_{j}Ref_{1,j}^{2} & \sum_{j}Ref_{1,j}\cdot Ref_{2,j} & \cdots & \sum_{j}Ref_{1,j}\cdot Ref_{n,j}\\ \sum_{j}Ref_{1,j}\cdot Ref_{2,j} & \sum_{j}Ref_{2,j}^{2} & \cdots & \sum_{j}Ref_{2,j}\cdot Ref_{n,j}\\ \cdots & \cdots & \cdots & \cdots \\ \sum_{j}Ref_{1,j}\cdot Ref_{n,j} & \sum_{j}Ref_{2,j}\cdot Ref_{n,j} & \cdots & \sum_{j}Ref_{n,j}^{2} \end{array}\right]$$

and an *n*-dimensional vector

(8) $${\vec{c}}^{T}=\left[\begin{array}{ccc} -\sum_{j}M_{j}\cdot Ref_{1,j} & -\sum_{j}M_{j}\cdot Ref_{2,j} & \cdots -\sum_{j}M_{j}\cdot Ref_{n,j} \end{array}\right].$$

The term in parentheses in Equation (6) is written in the standard form for QP, a linearly constrained quadratic optimization problem. Matrix *Q* and vector *c* of Equations (7) and (8) can be directly used as input parameters for standard QP algorithms. We used the so-called active set algorithm [44] to perform the optimization numerically.

## 3. Results and Discussion

### 3.1. Static Magnetic Offset Field Scan of Different Superparamagnetic Beads

**Figure 3:**
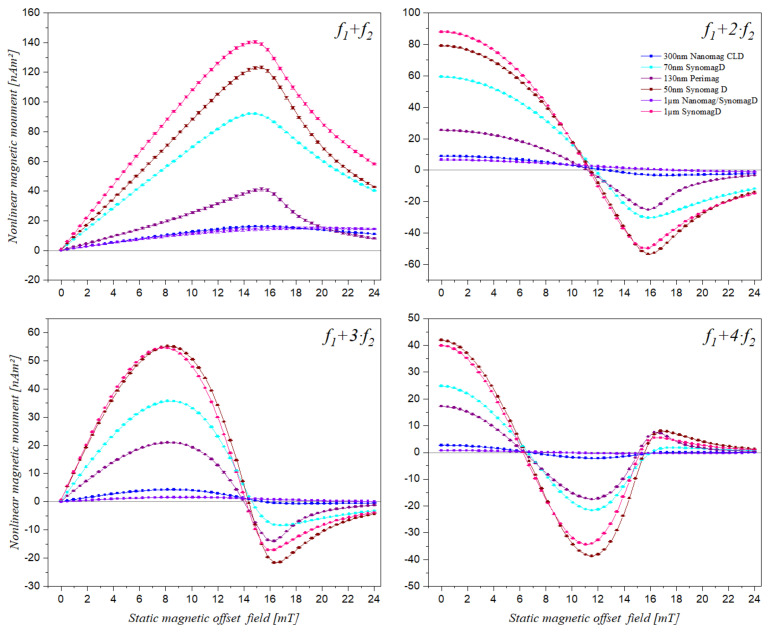
Measured nonlinear magnetic moment response (mean and standard deviation within point size) of different magnetic bead types at mixing frequencies *f*_1_ + *f*_2_, *f*_1_ + 2·*f*_2_, *f*_1_ + 3⋅*f*_2_ and *f*_1_ + 4⋅*f*_2_ over a static magnetic offset field range from 0 to 24 mT. The individual points are connected by lines for visual guidance.

Different MBs from different manufacturers mentioned in Section 2.1 were measured using the described setup, with an applied static magnetic offset field varying from 0 to 24 mT in steps of 0.48 mT. Figure 3 shows the nonlinear magnetic moment responses of different types of magnetic beds for mixing components *f*_1_ *+ f*_2_, *f*_1_ *+* 2·*f*_2_, *f*_1_ *+* 3⋅*f*_2_ and *f*_1_ *+* 4⋅*f*_2_. It can be seen that the mixing frequency component features varied for different types of beads. For example, the maximum peak measured for the sample 1 µm SynomagD occurred at about 14.88 mT at mixing component *f*_1_ *+ f*_2_, while for 300 nm Nanomag CLD, the maximum peak of the same component occurred at about 15.36 mT.

### 3.2. Effect of Amount of Beads on the Features of the Frequency Mixing Components

The amplitude of the frequency mixing signal was proportional to the amount of magnetic bead solution in a sample, this was utilized for the quantification of magnetic beads in different studies [14,15,16,17]. Since the observed features did also occur on the amplitude spectrum, their dependency on the amount of bead solution needed to be verified. For this purpose, different samples, each with a different amount of bead solution but of the same type (1 µm SynomagD), were prepared and measured over the specified range of static magnetic offset field. The features expressed by minima and maxima were determined by fitting a quadratic function to the corresponding region, and the zero crossings were determined through a linear fit to the corresponding region. The results are presented in Table 1. Statistical analysis was used to provide an appropriate assessment of the results. By looking at the relative standard deviation, we can see that the variation of the features among the samples having a different amount of beads did not exceed 1%. Thus, we conclude that there is no significant dependence of the signal features on the amount of bead solution.

### 3.3. Static Magnetic Offset Field Scan of Samples Containing Two Different Types of Superparamagnetic Beads

Utilizing the signatures of the beads on the mixing frequency features, five samples were prepared using two types of beads. The bead types selected for this experiment were 1 µm Nanomag/SynomagCLD, henceforth named A, and 1 µm SynomagD named B. The same bead types were used in previous work for particle distinction according to the phase of the frequency mixing response [30]. They are good candidates since they exhibit features that are much different from each other. Additionally, they are very interesting because they have both the same hydrodynamic diameter.

The reference samples of particles A and B were labelled as A and B, respectively, and the mixture samples were labelled as Mix1 to Mix3, the contents of which are given in Table 2.

**Figure 4:**
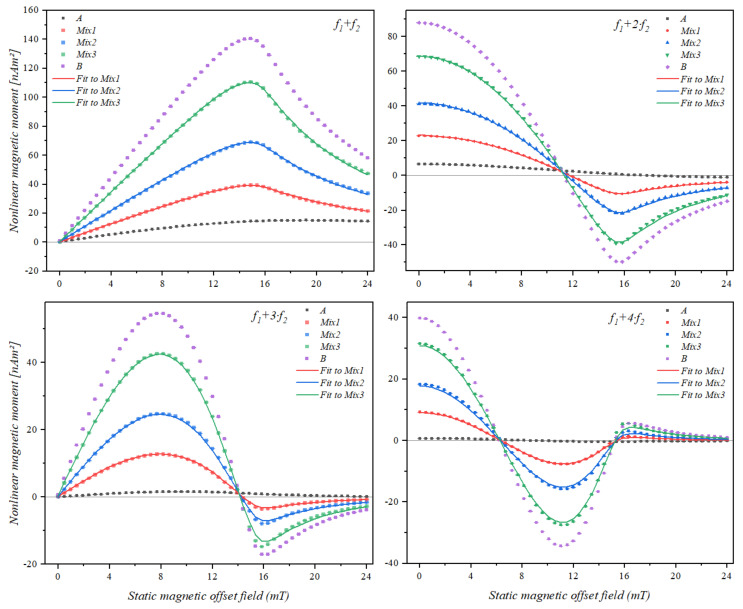
Nonlinear magnetic moment trace (mean and standard deviation within point size) of samples made from a mixture of two different types of magnetic beads over a static magnetic offset field range of 0 to 24 mT, for mixing frequencies *f*_1_ + *f*_2_, *f*_1_ + 2·*f*_2_, *f*_1_ + 3*⋅f*_2_ and *f*_1_ + 4*⋅f*_2_. A and B represent the samples containing pure bead A and pure bead B, respectively. Samples Mix1 to Mix3 contain different ratios of the two beads, they have been analyzed using the QP algorithm for determination of the contributions.

The trace of the nonlinear magnetic moment response of the binary mixture samples and the reference samples is shown in Figure 4.

The domination of the bead type B, which had a stronger response resulting in higher amplitude was also observed in the occurrence of the feature locations. Measurements of the mixture samples were analyzed using the QP algorithm for determination of the contributions. The optimization results are also shown in Figure 4.

**Figure 5:**
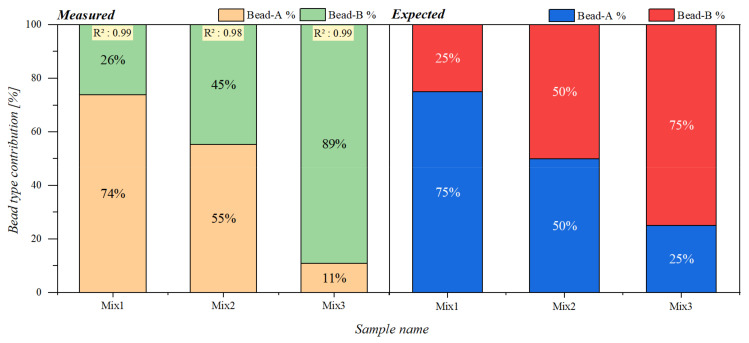
Bead type contributions for mixture samples containing beads A and B. The left chart presents the determined percentage of contributions from each reference bead type to the measured mixed samples. The right chart presents the expected contribution percentages of each bead type to the mixed samples.

Contributions of each pure sample (A and B) to the signal obtained from the mixed samples were then calculated using the quadratic programming approach interpolation mentioned in Section 2.6. The results are given in form of a percentage of the reference measurements in a bar chart representation in Figure 5 together with the expected ratios.

We can see that although the amplitudes of the two reference measurements (A and B) were very different from each other, the calculated contributions followed the prepared ratio patterns with a minimum deviation of 1% in case of Mix1 and a maximum deviation of 14% in case of Mix3.

To further investigate the effect of the magnetic response in the mixture samples, which also have been reported in [30], with respect to the phase of the frequency mixing signal, the particle type yielding a stronger response can be diluted. The amplitude adjustment was performed by diluting the 1 µm SynomagD (B) bead solution to 7% of its original concentration, resulting in a reduced amplitude response. A new set of samples were prepared using the same ratios expressed in Table 2 but with diluted stock. The samples containing the pure bead type were labelled as A* and B* and the mixture samples were labelled as Mix1*, Mix2*, and Mix3*, respectively.

**Figure 6:**
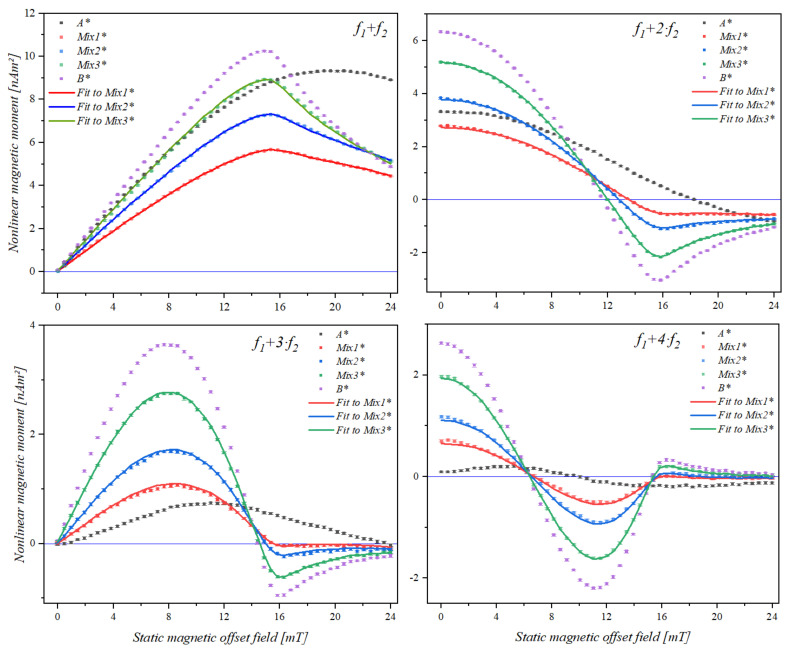
Nonlinear magnetic moment trace (mean and standard deviation within point size) of samples made from a mixture of two different types, 1 µm Nanomag/SynomagCLD (A*) and 1 µm SynomagD (B*), diluted to 7% of its original concentration. The responses of the mixture samples of the two bead types (Mix1* to Mix3*), prepared according to the ratios specified in Table 2, at mixing frequencies *f*_1_ + *f*_2_, *f*_1_ + 2*·f*_2_, *f*_1_ + 3*⋅f*_2_ and *f*_1_ + 4*⋅f*_2_ were recorded over a static magnetic offset field range from 0 to 24 mT. The response of the mixture samples was analyzed using the QP algorithm for determination of the contributions.

Figure 6 shows the magnetic field scan of the diluted set. Through investigating the features of the mixing frequency signals, we saw that although bead type B* is still highly affecting the location of the features, they become distinguishable due to the reduced strength of the signal from particle B. The locations of the features were determined using a quadratic fit for minima and maxima and a linear fit for the zero-crossings. The results are listed in Table 3.

By looking at the maxima of the mixing frequency signal *f*_1_ *+ f*_2_ for the two reference samples, we observed a deviation of 5.19 mT, and looking at mixture samples we saw that Mix3*, which contains more of the B* bead type had its feature occurring closer to that of B*.

Furthermore, looking at the features of *f*_1_ + 2·*f*_2_, in the case of sample A*, the zero-crossing occurred at 18.73 mT and had a deviation of 6.59 mT from B*. However, the minima of A* stayed undetermined since it occurred at magnetic fields beyond our scan range.

Comparing the two reference samples A* and B*, we saw that their features occurred within a relative standard deviation of at the lowest 21.23% in case of *f*_1_ *+ f*_2_ maxima and at the highest 36.38% in case of *f*_1_ + 4*·f*_2_ 1st zero-crossing. However, there were some regions where the feature remained undetermined because of the limited static magnetic offset field scan range.

On the other hand, when we examined the feature separation between the sample Mix3* and B*, we observed that the deviation gap was closing in such a way that we observed the maximum deviation of the 1st zero-crossing of *f*_1_ + 4·*f*_2_ and minimum deviation on the 2nd zero crossing of the same harmonic.

**Figure 7:**
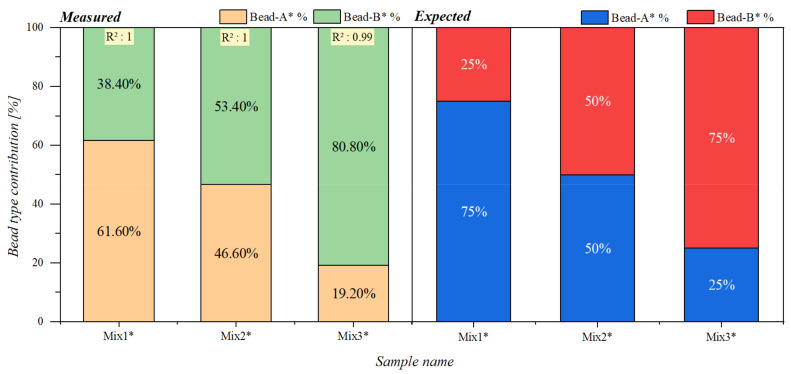
Bead type contributions for mixture samples containing beads A* and B*. The left chart presents the determined percentage of contributions from each reference bead type to the measured mixed samples. The right chart presents the expected contribution percentages of each bead type to the mixed samples.

The interpolation results in the case of these set of samples can be seen in Figure 7. They followed the expected pattern upon which the samples were prepared but with a maximum deviation of 13.4% in Mix1* sample.

The deviation occurring in both the diluted and undiluted mixture series could be due to the fact that the bead type 1 µm SynomagD (B and B*) dominated in the measured signals.

## 4. Conclusions

Characterization of different types of magnetic beads through frequency mixing magnetic detection using a static magnetic offset scanning technique revealed that the location of features occurring on the frequency mixing signals varies based on the type of magnetic beads. Furthermore, it was shown that the location of these features is independent of the amount of magnetic bead type in the sample, which makes them good type identifiers.

By investigating the effect of mixing two bead types in a sample, it was seen that the features deviated proportional to the ratio of bead types. Utilizing the information from these features together with the amplitude response of the beads which was used for quantification, the amount of each bead type in a sample could be determined. A simple straightforward approach is quadratic programming optimization to determine the best linear combination of the measured responses of the two reference beads. The estimated contributions were assessed and presented for both nondiluted and diluted mixture samples. The results showed a reasonable agreement with the initially prepared dilution with a maximum deviation of 14%. This approach is based upon the assumption that the bead types A and B did not interact with each other, and that the mixtures yielded a response that was a linear combination of the signals of each particle type. This assumption might not be true for very densely packed systems, which might require a more complex model.

## Figures and Tables

**Table 1:** The determining feature locations for samples with different amounts of magnetic bead solution.

Mixing Term	Bead Sample	1µSynD-6	1µSynD-8	1µSynD-10	Mean	Standard Deviation	Relative Std. Deviation
	Amount	6 µL	8 µL	10 µL			
*f*_1_ + *f*_2_	Maximum (mT)	14.48	14.65	14.66	14.60	0.10	0.7%
*f*_1_ + 2·*f*_2_	Zero (mT)	11.19	11.27	11.20	11.22	0.04	0.4%
	Minimum (mT)	15.49	15.57	15.63	15.57	0.07	0.4%
*f*_1_ + 3·*f*_2_	Maximum (mT)	7.75	7.89	7.89	7.84	0.08	1.0%
	Zero (mT)	13.99	14.05	14.14	14.06	0.07	0.5%
	Minimum (mT)	15.85	15.90	15.98	15.91	0.06	0.4%
*f*_1_ + 4·*f*_2_	1st Zero (mT)	6.19	6.24	6.30	6.24	0.06	0.9%
	Maximum (mT)	11.02	11.17	11.19	11.13	0.09	0.8%
	2nd Zero (mT)	15.02	15.06	15.03	15.04	0.02	0.1%
	Minimum (mT)	16.25	16.25	16.34	16.28	0.05	0.3%

**Table 2:** Amount of bead types used for the preparation of the reference samples (A and B) and the mixture samples Mix1 to Mix3. Bead type A resembles 1 µm Nanomag/SynomagCLD, and bead type B resembles 1 µm SynomagD.

		Mixture Samples				
		A (µL)	Mix1 (µL)	Mix2 (µL)	Mix3 (µL)	B (µL)
Bead Type	A	10	7.5	5	2.5	0
	B	0	2.5	5	7.5	10

**Table 3:** Location of the characteristic features of reference samples A* and B* and the mixture samples Mix1* to Mix3*. Some of the parameters remained undetermined due to the limited achievable static magnetic field, they are marked with “--”.

Mixing Term	Bead Sample	A*	Mix1*	Mix2*	Mix3*	B*
*f*_1_ + *f*_2_	Maximum (mT)	19.87	15.60	15.22	14.92	14.68
*f*_1_ + 2·*f*_2_	Zero (mT)	18.73	14.03	13.30	12.48	12.15
	Minimum (mT)	--	17.47	16.99	16.29	16.21
*f*_1_ + 3·*f*_2_	Maximum (mT)	11.02	8.27	8.10	7.79	7.78
	Zero (mT)	23.65	15.59	15.10	14.49	14.40
	Minimum (mT)	--	--	16.55	16.18	16.17
*f*_1_ + 4·*f*_2_	1st Zero (mT)	9.82	6.86	6.57	6.42	5.76
	Maximum (mT)	17.39	11.47	11.43	11.20	11.18
	2nd Zero (mT)	--	15.78	15.69	15.40	16.38
	Minimum (mT)	--	--	17.05	16.38	16.57

## Data Availability

The data presented in this study are available on request from the corresponding author.
